# Regulation of Fibroblast Cell Polarity by Src Tyrosine Kinase

**DOI:** 10.3390/biomedicines9020135

**Published:** 2021-02-01

**Authors:** Kazuo Katoh

**Affiliations:** Laboratory of Human Anatomy and Cell Biology, Faculty of Health Sciences, Tsukuba University of Technology, Tsukuba-city, Ibaraki 305-8521, Japan; katoichi@k.tsukuba-tech.ac.jp; Tel.: +81-29-858-9557

**Keywords:** cell motility, cell adhesion, polarity, c-Src, SFK

## Abstract

Src protein tyrosine kinases (SFKs) are a family of nonreceptor tyrosine kinases that are localized beneath the plasma membrane and are activated during cell adhesion, migration, and elongation. Due to their involvement in the activation of signal transduction cascades, SFKs have been suggested to play important roles in the determination of cell polarity during cell extension and elongation. However, the mechanism underlying Src-mediated polarity formation remains unclear. The present study was performed to investigate the mechanisms underlying Src-induced cell polarity formation and cell elongation using Src knockout fibroblasts (SYFs) together with an inhibitor of Src. Normal and Src knockout fibroblasts were also transfected with a wild-type c-Src, dominant negative c-Src, or constitutively active c-Src gene to analyze the changes in cell morphology. SYF cells cultured on a glass substrate elongated symmetrically into spindle-shaped cells, with the formation of focal adhesions at both ends of the cells. When normal fibroblasts were treated with Src Inhibitor No. 5, a selective inhibitor of Src tyrosine kinases, they elongated into symmetrical spindle-shaped cells, similar to SYF cells. These results suggest that cell polarity during extension and elongation may be regulated by SFKs and that the expression and regulation of Src are important for the formation of polarity during cell elongation.

## 1. Introduction

Cell polarity refers to the spatial, morphological, and structural asymmetry of cells and involves a characteristic spatial distribution of morphological, structural, and cellular components, i.e., the plasma membrane, cytoskeletal components, and sites of cell–cell adhesion (focal adhesions). Cell functions, such as transport, intracellular signaling, and mechanosensing, are dependent on the asymmetrical distribution of intracellular components. For example, epithelial cell polarity has an apical membrane–outer basal membrane orientation, and neural cell polarity is involved in the formation of two types of neurites, i.e., dendrites and axons [1]. Adhesive structures, adherens junctions, tight junctions, and focal adhesions allow the formation of two distinct compartments, i.e., the apical membrane that normally faces the external environment and the basolateral membrane that is connected to the extracellular matrix (ECM) of the basement membrane via integrins and their receptors.

Actin filaments are major components of actomyosin contractile systems in eukaryotic cells that regulate the direction of cell movement. Actin molecules switch between polymerization and depolymerization with the activation of Rho GTPases, a family of small G proteins, and their downstream molecules (the WASP/WAVE family and the Arp2/3 complex) [2,3]. Thus, stress fibers, lamellipodia, and pseudopodia, which control cell morphology and plasma membrane dynamics via actomyosin contractile systems, function in determining the polarization of cells [4].

When cells are cultured on a glass substrate, the plasma membrane begins to move in from the distal end to the leading edge of the cell [5]. Depolymerization of the actin cytoskeleton deforms the morphology of the cell membrane, and the focal adhesions between the extracellular matrix and intracellular proteins move forward to the leading edge of the cell while those at the rear of the cell are destroyed, forming a web-like structure when the cell moves, which results in the polarization of the moving cell [6].

The localization of receptors and adhesion molecules, such as integrins, is highly polarized in cultured cells when moving directionally [7]. Integrins are focal adhesion molecules that connect the inside of the cell and the extracellular matrix; they play essential roles in the regulation of membrane transport mechanisms [8,9]. Such dynamic changes in cell polarity during cell motility require changes in cytoskeletal components such as F-actin and microtubules, which are involved in the mechanism of membrane transport, closely associated with the membranous cytoskeletal systems described above [9].

Endocytosis of integrins occurs at the leading edge of cells, but it has not been observed at the trailing edge. As they are the molecules that control this localization, the turnover of focal adhesions by endocytosis or exocytosis of integrins is necessary for cell movement. The organization of focal adhesions is controlled by focal adhesion kinase (FAK) and its substrates, including members of the Src family of protein tyrosine kinases (SFKs) [10].

The cell–substrate interface, referred to as a focal adhesion or adhesion plaque, plays essential roles in many biological behaviors, including cell migration, wound healing, and angiogenesis. These areas are composed of typical focal adhesion constituent proteins such as vinculin, paxillin, talin, alpha-actinin, and integrin [11,12,13,14,15]. Some signal transduction proteins, such as FAK, cellular Src (c-Src), and Rho A, are also colocalized with these constituent proteins, in close association with stress fibers and focal adhesions [16,17,18]). These observations strongly suggest that the focal adhesions play roles in transferring certain migration and polarization signals from the external environment to the inside of the cell. Focal adhesions recognize the boundary between the plasma membrane and extracellular matrix proteins such as fibronectin and vitronectin, and focal adhesions also determine cell orientation and polarity during cell movement [19]. 

The SFKs are a family of nonreceptor-type protein tyrosine kinases associated with the plasma membrane, which play roles in cell–matrix and cell–cell adhesion and are present in endosomal vesicles. Src mediates signaling by a variety of receptors [20,21], and activated Src induces cell transformation in vitro [22,23,24]. For example, Src expression and activity are elevated in many human epithelial cancers [25]. The first 16 N-terminal amino acids of Src are required for membrane binding [26], and the subsequent 17–84 amino acids constitute a unique domain. This is followed by the SH3 and SH2 domains, which are connected by a short linker, with another linker connecting the SH2 domain to the kinase domain, which is required for most of the biological functions of Src [26]. Tyr527 (or Tyr530 in human Src, Tyr534 in mouse, equivalent to Tyr527 in chicken) undergoes inhibitory phosphorylation by the C-terminal Src kinase. In the inactive or “closed” form of Src, the SH2 domain interacts with pTyr527 and places the SH3 domain in the correct position to interact with the polyproline type II helix of the kinase-SH2 linker region, thus inactivating the conformational change in the N-terminal domain of the kinase. Activation can occur due to dephosphorylation or mutation of Tyr527 or by binding of the activating ligand to the SH2 or SH3 domain [22,23,24,27]. Mutation of Tyr527 in c-Src that prevents phosphorylation causes enzymatic activation [28].

In normal cells, c-Src is involved in a wide range of physiological functions, including cell proliferation, migration, construction of the cytoskeleton, and interaction with the extracellular matrix. Src was shown to translocate to focal adhesions at the cell periphery [29], where it undergoes activation-dependent association with focal adhesions and the associated stress fibers [30]. FAK contains a defined focal adhesion targeting domain and an Src binding site created by FAK autophosphorylation [31,32]. Src exists in an inactive form, phosphorylated by the specific regulator C-terminal Src kinase (CSK), and is activated in response to a diverse array of extracellular stimuli, such as growth factors and extracellular matrix [27]. CSK is a kinase that phosphorylates Tyr527, the negative regulatory site of c-Src [28]. Tyr527-phosphorylated c-Src induces gene expression through activation of the MAPK pathway and induces focal adhesions and other types of cytoskeletal reorganization by activating Rho GTPases. These observations provide evidence that the tyrosine kinase activity of the Src family at focal adhesions regulates the metabolic turnover of focal adhesions during cell motility, resulting in the formation of cell polarity. As a result, Src exerts diverse physiological functions, such as activation of cell adhesion and motility, differentiation, proliferation, survival, and even induction of transformation [29,33]. The present study was performed to examine the effects of the expression of activated or dominant negative forms of the c-Src gene in fibroblasts to gain insight into the role of Src tyrosine kinase in cell motility, especially in the establishment of cell polarity.

Fibroblasts were treated with Src Inhibitor No. 5, a selective inhibitor of Src tyrosine kinases, to block Src activation and allow us to investigate its influence on the physiological characteristics of the cells and elucidate the mechanisms underlying polarized elongation. Normal fibroblasts and Src family knockout cells [34] (SYF cells; ATCC, Manassas, VA) were also transfected with constructs encoding wild-type c-Src (WT c-Src), constitutively active c-Src, and dominant negative c-Src genes to analyze the changes in cell morphology. In this study, SYF cells transfected with WT c-Src showed an elongated morphology while extending pseudopodia similar to normal fibroblasts. Normal fibroblasts transfected with the dominant negative c-Src mutant gene showed symmetrical spindle-shaped morphology. Normal fibroblasts treated with the c-Src-specific inhibitor, Src Inhibitor No. 5 (Biaffin, Kassel, Germany), adopted a symmetrical spindle shape similar to SYF cells. These observations suggest that SFKs regulate polarity formation during cell extension and elongation and that the expression and regulation of at least one member of the SFKs, c-Src, are important for polarity formation during cell elongation.

## 2. Materials and Methods

### 2.1. Cell Culture

Fibroblasts (3T3 cells; NIH, Bethesda, Rockville, MD, USA) or SFK-knockout mouse fibroblasts (SYF cells; ATCC) [34] were cultured in a 1:1 mixture of Dulbecco’s modified Eagle’s medium (DMEM) and a nutrient mixture (Gibco, Grand Island, NY, USA), pH 7.4, containing 50 units/mL of penicillin, 50 µg/mL of streptomycin, and 10% fetal bovine serum (Gibco). The cells were maintained at 37 °C in a humidified, 5% CO_2_ atmosphere. Cells were cultured overnight on glass-bottomed culture dishes of 35 mm diameter (Matsunami Glass, Tokyo, Japan) and used in the experiments.

### 2.2. Immunofluorescence Microscopy

Cultured cells were fixed with 1% paraformaldehyde in PBS for 30 min and permeabilized by treatment with 0.05% Triton X-100 in PBS for 5 min. The fixed cells were incubated with 10% normal goat serum for 30 min at room temperature and then stained with an antibody against vinculin (Sigma, St. Louis, MO, USA) as a marker of focal adhesions for 60 min. After washing in PBS for 20 min, the fixed specimens were incubated with fluorescein-conjugated anti-mouse IgG. Samples were then observed by conventional epifluorescence microscopy or phase-contrast microscopy (Olympus, Tokyo, Japan).

### 2.3. SFK Inhibitor

The SFK inhibitor, Src Inhibitor No. 5 (Biaffin), is one of several Src inhibitors that belong to the quinazoline class of selective inhibitors of c-Src and has an IC_50_ of 10 nM in vitro [35,36,37]. Fibroblasts were seeded onto dishes and cultured for 24 h, followed by incubation with Src Inhibitor No. 5 at a concentration of 10 μM. 

### 2.4. Transfection of pUSEamp-WT c-Src, pUSEamp-Dominant Negative c-Src (K295M/Y527F), and pUSEamp-Constitutively Active c-Src (Y527F) Vectors

Dominant negative c-Src (K295M/Y527F) and constitutively active c-Src (Y527F; chicken c-Src residue numbering) were generated by site-directed mutagenesis using a KOD-plus mutagenesis kit, in accordance with the manufacturer’s protocol (Toyobo, Tokyo, Japan), and cloned into a cytomegalovirus (CMV) promoter-driven pUSEamp-WT Src expression vector (Upstate Biotechnology, Lake Placid, NY, USA). WT Src or dominant negative c-Src (K295M/Y527F) was inserted into peGFP-C1 (Clontech, Palo Alto, CA, USA). eGFP-fused dominant negative c-Src (K295M/Y527F) and WT c-Src were transfected into fibroblasts using Tfx-50, in accordance with the manufacturer’s protocol (Promega, Madison, WI, USA). pUSEamp-dominant negative c-Src (K295M/Y527F), constitutively active c-Src (Y527F), or WT c-Src was also transfected into fibroblasts using Tfx-50 (Promega), in accordance with the manufacturer’s protocol. Cells transfected with eGFP-C1 or pUSEamp(−) alone did not show any differences in morphology from normal fibroblasts. Neomycin-resistant cells were selected by growth in 400 μg/mL G-418 and maintained in 200 μg/mL G-418. G-418 was removed prior to the experiments. Control cells were transfected with vector alone, cloned, and treated in a manner similar to that described for the experimental cells. Transfected cells were plated on glass-bottomed culture dishes and placed on a temperature-controlled stage at 37 °C (Matsunami, Tokyo, Japan). The cell–substrate interface was examined by conventional phase-contrast microscopy and epifluorescence microscopy (Olympus).

### 2.5. Calculation of the Vertical Axis of the Cell and the Aspect Ratio

The vertical axis and aspect ratio of 50 cells were measured using open-source Fiji image analysis software [38]. Statistical analyses were performed using Excel (Microsoft, Redmond, WA, USA). Unpaired two-tailed Student’s *t*-test was used to compare the means between the two groups. All statistical tests were two-sided, and *p* < 0.05 was taken to indicate statistical significance.

## 3. Results

SFKs are nonreceptor tyrosine kinases that play key roles in the regulation of signal transduction. SFK activation and protein levels are elevated in various types of cancer, and there has been a great deal of research regarding the regulation of Src kinase activity. SFKs consist of several proteins, i.e., Src, Fyn, Yes, Fgr, Lck, Hck, Blk, Lyn, Frk, and Yrk, that interact with the intracellular domains of growth factors/cytokine receptors, G protein-coupled receptors (GPCRs), and integrins [39,40,41,42]. Members of the SFK family have similar domain structures, and several small molecule inhibitors that show selectivity for SFKs are available. 

**Figure 1:**
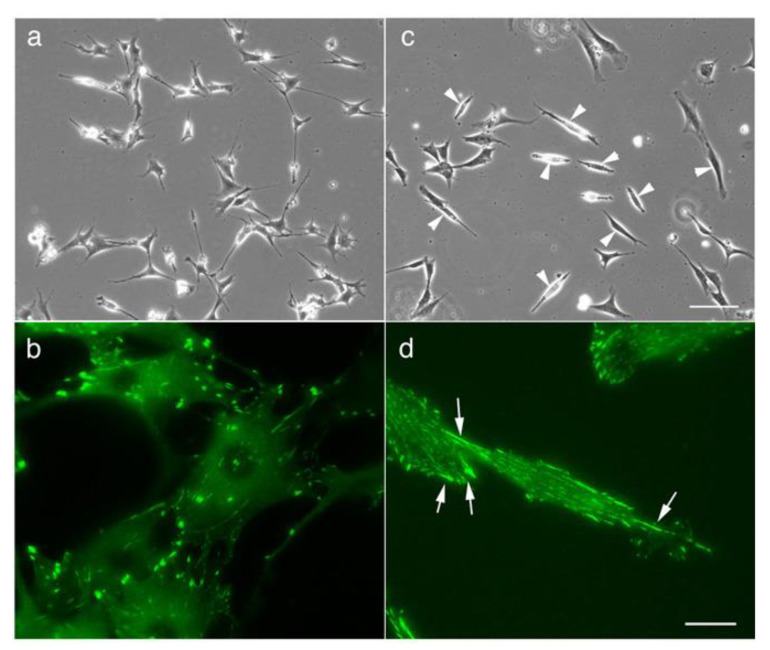
Morphology of normal 3T3 cells and Src knockout fibroblast (SYF) cells (c-Src, c-Yes, and Fyn knockout cells), as observed by phase-contrast microscopy. (**a**) The morphology of normal fibroblasts. (**b**) Fluorescence microscopy showing focal adhesions stained with anti-vinculin antibody. (**c**) When SYF cells were cultured on a glass substrate, they first showed a pancake-like morphology and then adopted a symmetrical spindle shape (arrowheads). (**d**) In this process, focal adhesions were formed at both ends of the cells, and a relatively small adhesive patch-like structure was observed at the center of the cells (arrows). (**a**,**c**) Phase-contrast microscopy. (**b**,**d**) Fluorescence microscopy showing focal adhesions stained with anti-vinculin antibody. Scale bars: (**a**,**c**), 100 μm; (**b**,**d**), 20 μm.

When cultured on a glass substrate, SYF cells first adopted an extended pancake shape and then spread out into symmetrical spindle-shaped cells (Figure 1c, arrowheads; compare to Figure 1a for normal fibroblasts adhering to the coverslip). In this process, typical focal adhesions are formed at both ends of the cells, and many relatively small adhesive patch-like structures are observed at the center of the cells (Figure 1d, arrow; compare to Figure 1b for normal fibroblasts adhering to the coverslip).

**Figure 2:**
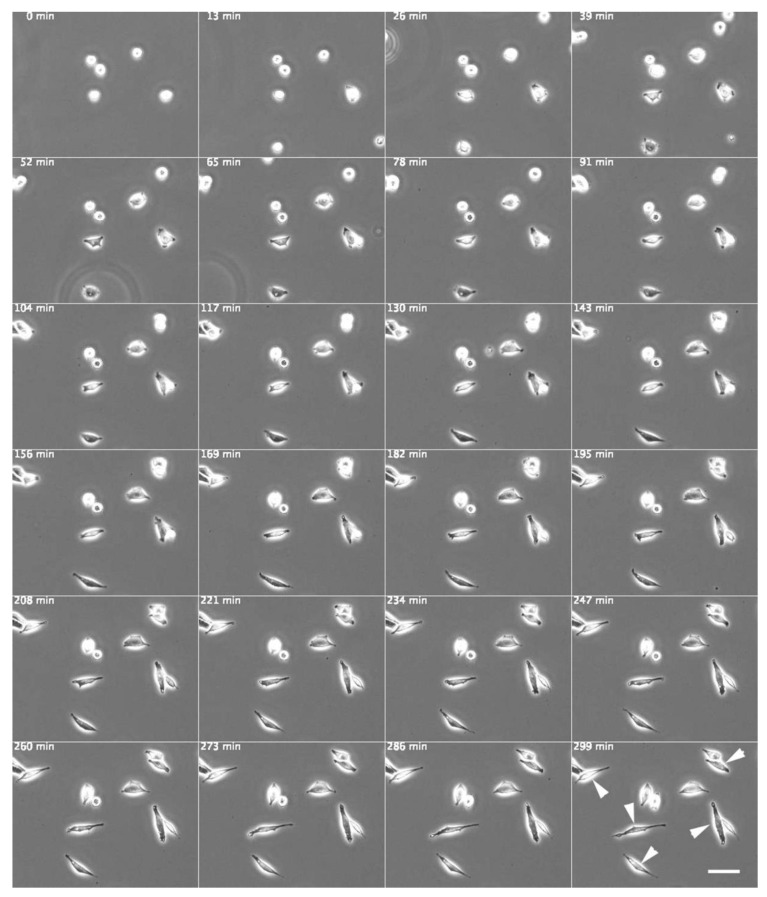
Normal fibroblasts cultured on a glass substrate in medium containing c-Src inhibitor (Src Inhibitor No. 5). Normal fibroblasts cultured in a medium containing 10 μM Src Inhibitor No. 5, a c-Src inhibitor, adhered to the glass substrate and then showed symmetrical spindle-like extension (arrowheads). The morphology was similar to that of SYF cells stretched on the glass substrate (see Figure 1c). Phase-contrast microscopy time-lapse images. The numbers at the top left indicate the number of minutes since the start of incubation. Scale bar, 100 μm. See also Video S1.

Cells were treated with Src Inhibitor No. 5, a selective inhibitor of Src tyrosine kinases, to investigate its influence on the physiological characteristics of fibroblasts and elucidate the mechanisms underlying polarized cell elongation. Normal fibroblasts cultured in medium containing 10 μM Src Inhibitor No. 5 first adhered to the glass substrate and then showed a symmetrical spindle-like extension (Figure 2, arrowheads), similar to the morphology of SYF cells stretched on the glass substrate (see Figure 1c), as shown in the time-lapse phase-contrast microscopy images (Video S1).

**Figure 3:**
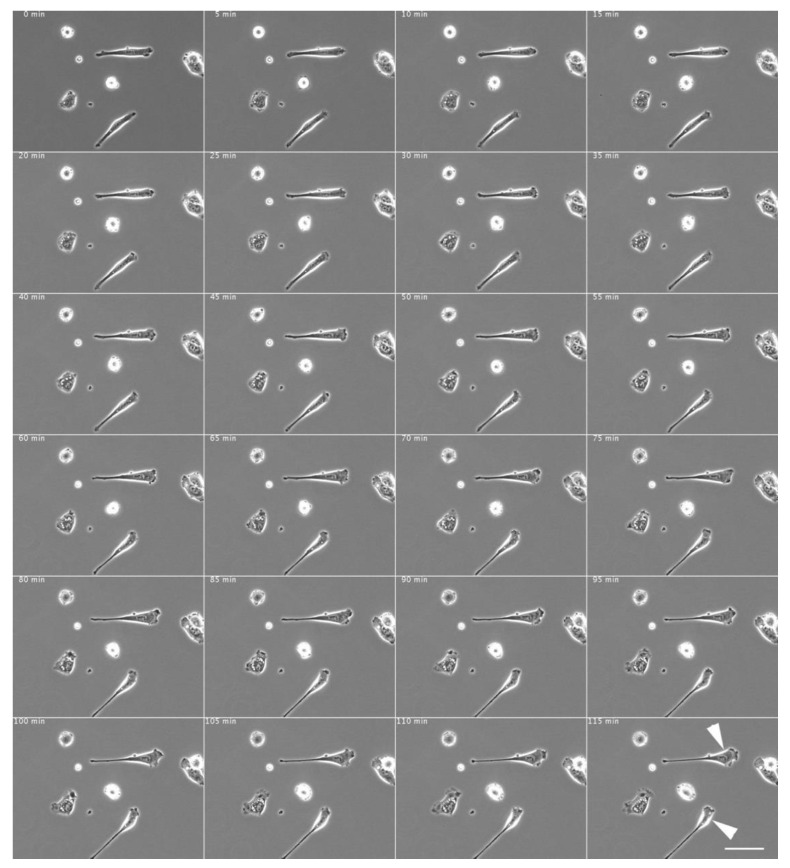
Morphological changes in normal fibroblasts after removal of c-Src inhibitor. After washout of the c-Src inhibitor with inhibitor-free culture medium, the elongated fibroblasts began to migrate in one direction, extending their pseudopods and eventually showing typical fibroblast morphology (arrowheads). The medium of the cells shown in Figure 2 was replaced with normal culture medium. The time intervals are shown in minutes at the top left corner of each time-lapse phase-contrast microscopy image. Scale bar, 100 μm. See also Video S2.

After washing the c-Src inhibitor-treated cells (shown in Figure 2) with an inhibitor-free culture medium, the symmetrically stretched fibroblasts began to show directed migration and extended pseudopodia and eventually exhibited typical fibroblast morphology (Figure 3; see also Video S2).

**Figure 4:**
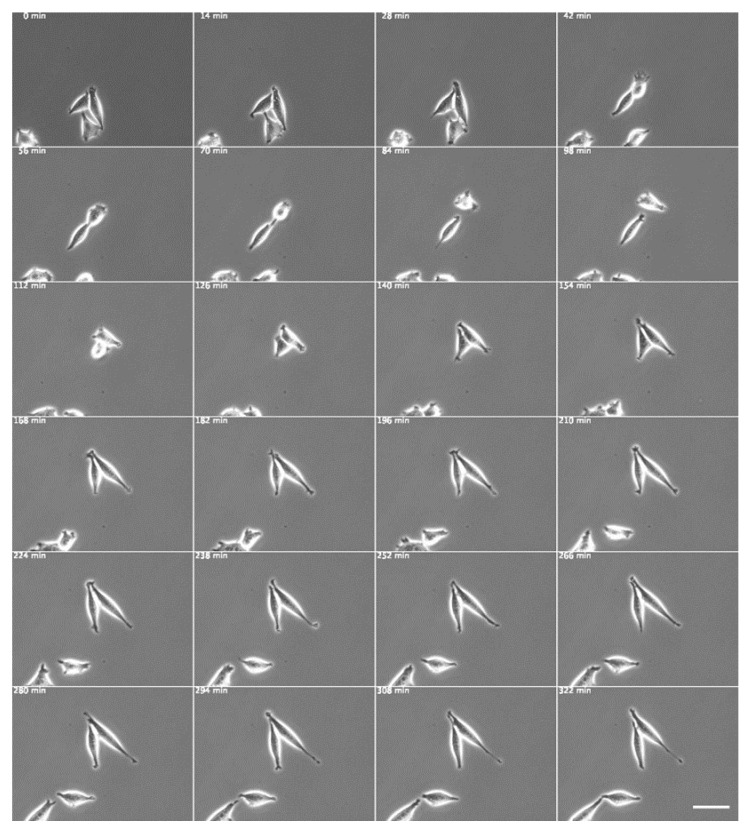
Normal fibroblasts expressing the dominant negative c-Src gene. The dominant negative c-Src gene (pUSEamp-dominant negative c-Src) was expressed in normal fibroblasts, and living cells were recorded under phase-contrast microscopy. The time intervals are shown in minutes at the top left corner of each time-lapse phase-contrast microscopy image. Scale bar, 100 μm. See also Video S3.

**Figure 5:**
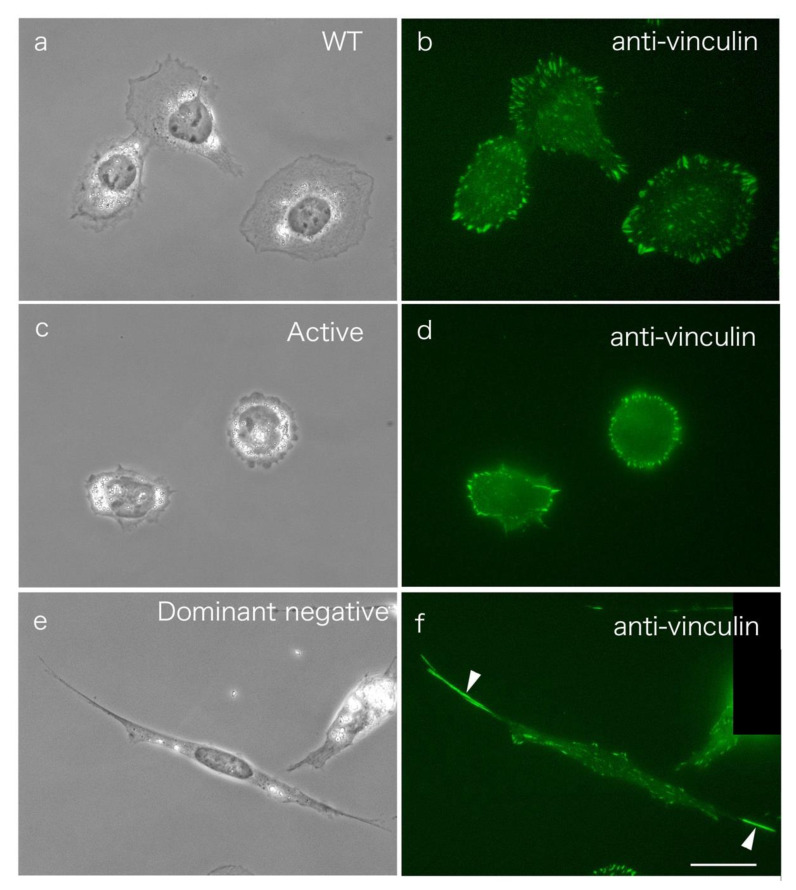
Normal fibroblasts expressing WT c-Src, constitutively active c-Src, and dominant negative c-Src genes. pUSEamp-WT c-Src (**a**,**b**), pUSEamp-constitutively active c-Src (**c**,**d**), and pUSEamp-dominant negative c-Src (**e**,**f**) were transfected into normal fibroblasts, and fixed cells were recorded under phase-contrast microscopy. Cells expressing the dominant negative c-Src gene were elongated at both poles in a symmetrical cone shape (**e**,**f**). (**c**) The cells expressing constitutively active c-Src show a pancake-like morphology. (**a**,**c**,**e**) Phase-contrast microscopy. (**b**,**d**,**f**) Fluorescence microscopy of cells stained with anti-vinculin antibody. Figure 1b shows normal fibroblasts stained with anti-vinculin antibody as a control. Scale bar, 20 μm.

Next, we examined the effects of transfection of cultured fibroblasts with a dominant negative Src expression vector (Figure 4 and Figure 5). Mutation of Lys295 in the catalytic site to methionine (K295M) inactivates Src kinase activity [43,44]; studies on mutant cells have indicated that both splicing and transport activities require the kinase activity of Src [45]. An Src mutant containing both Src K295M/Y527F mutations was also generated and was shown to have an open conformation and no kinase activity but to retain SH2 and SH3 binding activity due to the lack of interaction with intramolecular SH2-pTyr527 [46,47]. A constitutively active Src mutant was also generated by mutating the inhibitory Tyr527 to phenylalanine (Y527F). The dominant negative c-Src gene (pUSEamp-dominant negative c-Src) was expressed in normal fibroblasts, and living cells were recorded under phase-contrast microscopy (Figure 4). Cells expressing the dominant negative c-Src gene were elongated at both poles in a symmetrical cone shape.

To examine c-Src expression in the polarization of cells, we transfected the constitutively active or dominant negative form of c-Src into normal fibroblasts (Figure 5). Here, pUSEamp-WT c-Src (Figure 5a,b), pUSEamp-constitutively active c-Src (Y527F; Figure 5d,e), and pUSEamp-dominant negative c-Src (K295M/Y527F; Figure 5d,e for a single image of the fixed cells) were transfected into normal fibroblasts. Some cells were stained with an antibody to vinculin as a marker of focal adhesions (Figure 5b,d,f). The cells expressing WT c-Src showed almost the same morphology as normal fibroblasts (Figure 5a,b), while cells expressing dominant negative c-Src showed a symmetrical cone shape (Figure 5e,f; see also Figure 4). Focal adhesions were not significantly different from those of normal fibroblasts in cells expressing normal c-Src but were formed at both ends of the cells expressing dominant negative c-Src (Figure 5e, phase-contrast microscopy; Figure 5f, staining for vinculin). The cells expressing constitutively active c-Src showed a pancake-like morphology (Figure 5c), and vinculin-positive focal adhesions were observed mainly at the cell periphery but not in the center of the cell (Figure 5d). 

**Figure 6:**
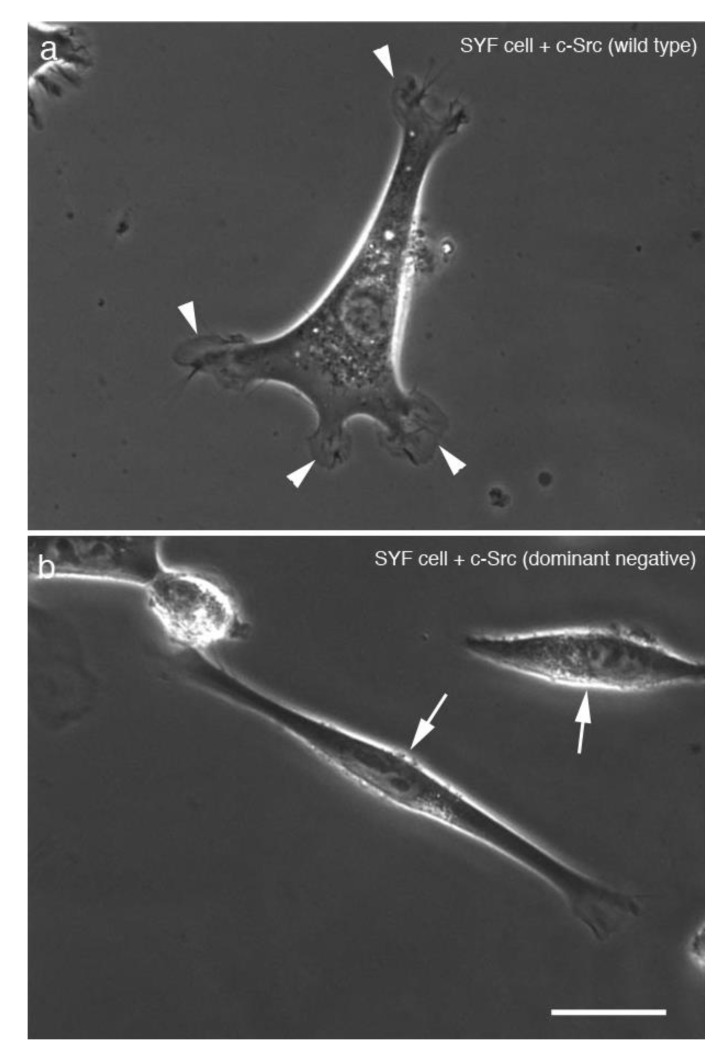
SYF cells expressing eGFP-fused wild-type (WT) c-Src and eGFP-fused dominant negative c-Src genes. SYF cells expressing (**a**) eGFP-fused WT c-Src and (**b**) eGFP-fused dominant negative c-Src were observed by phase-contrast microscopy. Cells transfected with (**a**) eGFP-fused WT c-Src were elongated with pseudopodia (arrowheads) similar to normal fibroblasts, whereas (**b**) cells expressing eGFP-fused dominant negative c-Src showed an elongated symmetrical cone shape (arrows). Scale bar, 20 μm.

SYF cells expressing eGFP-fused WT c-Src (Figure 6a) and eGFP-fused dominant negative c-Src (Figure 6b) were examined by phase-contrast microscopy. Transfection of the cells with each eGFP-fusion gene construct was examined by fluorescence microscopy, and photographs were taken under phase-contrast microscopy. Cells expressing eGFP-fused dominant negative c-Src had a symmetrical shape (Figure 6b). SYF cells cultured on a glass substrate were extended with a symmetrical slim spindle-like structure. When transfected with a vector carrying the wild-type c-Src gene, SYF cells were extended with pseudopodia and showed normal fibroblast-like morphology. 

**Figure 7:**
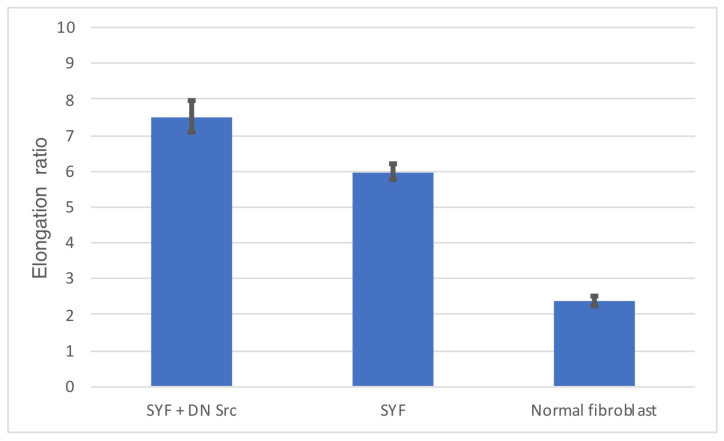
Elongation ratio of the vertical axis to the long axis.

Normal fibroblasts transfected with a vector encoding dominant negative c-Src adhered to the glass substrate and began to extend in both polar regions, eventually becoming spindle-shaped cells. The mean ± standard error of the mean (SEM) aspect ratio of the vertical axis to the long axis of the SYF cells transfected with dominant negative c-Src was 7.51 ± 0.43 (*n* = 50). The aspect ratio of SYF cells was 5.98 ± 0.24, while the aspect ratio of normal fibroblasts was 2.38 ± 0.13 (Figure 7). 

SYF cells cultured on a glass substrate adopted a symmetrical spindle shape. SYF cells transfected with a vector carrying WT c-Src became elongated, had extended pseudopodia, and showed almost the same morphology as normal fibroblasts. Normal fibroblasts transfected with the vector carrying the dominant negative c-Src gene adopted a symmetrical spindle shape. On treatment with the specific c-Src inhibitor, Src Inhibitor No. 5, normal fibroblasts were elongated in a similar manner to SYF cells, with focal adhesions formed at both ends of the elongated cells. Following washout of the c-Src inhibitor, the pseudopodia were actively extended, and the cells returned to a morphology that was almost identical to normal fibroblasts. These observations suggest that polarity formation during cell extension and elongation is regulated by the SFKs and that the expression and regulation of at least one member of the family, c-Src, are important for polarity formation during cell elongation.

## 4. Discussion

Signal transduction mechanisms in cells involve tyrosine phosphorylation for activation or inactivation of specific proteins. The levels of tyrosine phosphorylation reflect the local levels of signal transduction activity. Phosphotyrosine proteins are highly accumulated at the sites of focal adhesion in cells in culture, reflecting the involvement of these sites in signal transduction. SFKs are membrane-bound, nonreceptor tyrosine kinases that function as important signaling intermediates in the regulation of cell proliferation, differentiation, apoptosis, migration, and metabolism [21,25,48].

Due to their role in regulating cellular adhesion, the turnover of integrins by endocytosis or exocytosis is necessary for cell movement [49]. These processes seem to be controlled by FAK and its substrates [50], including SFKs [51]. SFK is a family of oncogenes that were initially discovered in association with cancer. Tumors in chickens were shown to be caused by the Rous sarcoma virus oncogene, v-Src, which is similar to the typical cellular protein, c-Src. Unlike c-Src, v-Src is constitutively active as it lacks the C-terminal inhibitory phosphorylation site (Y527) [52]. The c-Src protein is a signaling molecule that has important roles in controlling cell growth, proliferation, and/or motility.

Stress fibers are a contractile apparatus that can generate isometric tension in cells, which is possible because both ends of the stress fibers are anchored to the substrate via focal adhesions [17]. Cell motility seems to be regulated by the crosstalk between Rho, Rac, Ras, and/or Cdc42. Although the mechanisms underlying the regulation of cell polarity are still unknown, the results of the present study indicate the roles of activation and deactivation of c-Src in the control of cell polarity. Normal fibroblasts transfected with dominant negative c-Src showed greater cell elongation than control cells.

In this study, SYF cells transfected with dominant negative c-Src retained an elongated morphology. In addition, WT c-Src-transfected SYF cells showed almost the same morphology as normal fibroblasts. When the cell migrates, the leading edge shows a filopodium-like structure. Migrating cultured cells have polarity, with a leading edge at the front of the cell and a trailing edge at the rear of the cell. However, the mechanism by which the leading edge and the trailing edge are specified is still unclear. The results of the present study suggest that deactivation of c-Src results in an elongated morphology and a symmetrical cell shape [19].

SFKs are translocated to the sites of cell adhesion [53]. Previous studies have shown that Src kinase activity influences cell proliferation and cell migration [54,55,56]. Moreover, the results presented here suggest that the kinase activity of SFKs plays a role in the regulation of symmetrical elongation of fibroblastic cells under physiological conditions.

SYF cells showed no consistent differences in the actin cytoskeleton in comparison to WT controls. Focal adhesions form in the absence of tyrosine phosphorylation of focal adhesion-associated proteins, and SYF cells show reduced motility [34]. In this study, the reintroduction of WT c-Src into SYF cells restored the defective motility and bipolarity of the fibroblastic cells. SYF cells formed focal adhesions, which showed a typical arrowhead-like appearance and were present in the same numbers as in normal fibroblasts; no differences in the number of stress fibers were observed [57]. The present study demonstrates that SYF cells and normal fibroblasts transfected with dominant negative c-Src will show reduced cell motility, consistent with previous reports. Moreover, fibroblasts transfected with dominant negative c-Src showed polarized movement, with a symmetrical cell shape. These results indicate that the deactivation of c-Src plays a role in cell motility, resulting in cell polarization. With regard to fibroblastic cell proliferation, the activity of Src is physiologically important to the polarized elongation of the cell.

The initiation of Src tyrosine kinase signal transduction pathways leads to the proliferation and activation of fibroblasts, which deposit extracellular matrix into the surrounding connective tissue. Fibrosis, defined by the accumulation of excess extracellular matrix components, is a pathological feature of most chronic inflammatory diseases. The results of the present study show that Src tyrosine kinase is a candidate molecule involved in the regulation of fibroblast elongation and polarization. The interaction of Src with integrin αV is required for integrin αV-mediated Src activation and subsequent fibroblast migration [58]. The interaction of SFKs and integrins plays critical roles in the development of lung fibrosis [59,60,61], liver fibrosis [62,63], and chronic kidney disease [64]. SFKs seem to play critical roles in the development of lung fibrosis, and the signaling involved may represent a novel opportunity to target fibrotic diseases [58]. The pathogenesis of these diseases is not fully understood, but they do appear to be associated with alterations in fibroblast migration and excessive matrix deposition [65,66]. Fibroblast migration and proliferation are tightly controlled processes [67,68]. Protein-kinase-regulated cell migration is involved in the development of lung fibrosis [66]. Src tyrosine kinase regulates focal adhesion kinase activation and seems to cause cell elongation [69]. However, the involvements of Src tyrosine kinase in fibroblast migration and proliferation and in lung fibrosis have yet to be explored in detail. Treatment with the Src inhibitor, PP2, was reported to significantly reduce fibroblast migration stimulated by platelet-derived growth factor-BB (PDGF-BB) and to reduce lung fibrosis in mice in vivo [58]. The results of the present study indicate that c-Src tyrosine kinase is involved in fibroblastic polarized cell elongation and symmetrical cell shape changes. It has been suggested that Src tyrosine kinases are involved in the pathogenesis of renal fibrosis, and the selective Src tyrosinase inhibitor PP1 may inhibit fibrosis and have therapeutic potential for the treatment of CKD chronic kidney disease [70]. The findings outlined above suggest that SFKs play roles in the development of fibrosis that is related to the proliferation and elongation of fibroblasts.

The mechanism by which the cell determines the direction of migration is still unclear. The direction of migration seems to be determined randomly, according to the orientation of stress fibers in cultured cells. The position of the Golgi apparatus appears to be critical in specifying various aspects of cell migration [71]. Disruption of the Golgi apparatus, even under conditions where the cytoskeleton remains intact, results in failure of cell polarization and inhibition of cell migration [71]. The microtubule organization center (MTOC) and its associated microtubules determine the position of the Golgi apparatus [72], which is thought to facilitate polarized secretion of the plasma membrane at the leading edge of the cell [73]. The positions of the Golgi apparatus and MTOC have been suggested to be involved in determining the direction of cell migration. However, as focal adhesions represent the footholds of migrating cells, elongation and determination of polarization seem to be determined to a greater extent by cytoskeletal components than the Golgi and/or MTOC associated with focal adhesions.

In this study, cells transfected with a dominant negative c-Src construct showed a symmetrical shape, suggesting that the deactivation of c-Src causes bidirectional elongation. Moreover, the formation of pseudopodia at the leading edge of the cell was reduced. Well-developed focal adhesions were detected at both ends of the leading edge of these cells. Random migration was inhibited in cells transfected with the dominant negative c-Src construct. The Arp 2/3 complex is involved in the regulation of the actin-containing cytoskeleton in pseudopodia at the leading edge of the cell. Arp 2/3 interacts with the small rho-type GTPase Cdc42, and, thus, Arp 2/3 may align actin filaments to the plasma membrane through the activity of Cdc42 [3,74]. Deactivation of c-Src resulted in a lack of pseudopodia at the leading edge of the cell (Figure 5), reflecting the disorganization of pseudopodia in both c-Src-deficient cells and cells with deactivated c-Src. The above observations suggest that the deactivation of c-Src also causes Cdc42-dependent organization of the Arp 2/3 meshwork in pseudopodia structures. 

SYF cells lacking the SFK members Src, Yes, and Fyn, cultured on glass substrates, were observed to elongate into symmetrical spindle-shaped cells. The introduction of the WT c-Src gene into SYF cells resulted in the elongation and extension of pseudopodia, and they adopted a morphology that was similar to normal fibroblasts. Normal fibroblasts transfected with a construct carrying the dominant negative c-Src gene adopted a symmetrical spindle shape. Treatment of normal fibroblasts with the c-Src-specific inhibitor, Src Inhibitor No. 5, resulted in symmetrically shaped cells, similar in morphology to SYF cells. Focal adhesions were formed at both ends of the elongated cells. Following washout of the c-Src inhibitor, the pseudopodia were actively extended, and the cells returned to a morphology that was almost identical to normal fibroblasts. Other Src family inhibitors caused the cells to adhere to the glass substrate but not to adopt the spindle-shaped morphology. These observations address the question of how the symmetrically elongated features of the cell are organized. The results of the present study indicate that cells transfected with the dominant negative c-Src gene or incubated with a c-Src inhibitor will first attach to the glass substrate with a pancake-like morphology, after which they elongate along both poles and adopt a symmetrical shape within 2 h. Inactivation of c-Src results in highly elongated cells, both ends of which are slender and narrow in shape. 

The results of the present study suggest that polarity formation during cell extension and elongation may be regulated by the Src family and that the expression and regulation of at least one member of the family, c-Src, are important for polarity formation during cell elongation.

## Data Availability

The data presented in this study are available on request from the corresponding author.

## Supplementary Materials

The following are available online at https://www.mdpi.com/2227-9059/9/2/135/s1, Video S1: Normal fibroblasts cultured on a glass substrate in medium containing c-Src Inhibitor No. 5., Video S2: Morphological changes in normal fibroblasts after removal of c-Src inhibitor No. 5., Video S3: Normal fibroblasts expressing the dominant negative c-Src gene.
